# Perturbation of IIS/TOR signaling alters the landscape of sex-differential gene expression in Drosophila

**DOI:** 10.1186/s12864-018-5308-3

**Published:** 2018-12-10

**Authors:** Rita M. Graze, Ruei-Ying Tzeng, Tiffany S. Howard, Michelle N. Arbeitman

**Affiliations:** 1 Department of Biological Sciences, Auburn University, 101 Rouse Life Sciences building, Auburn, AL 36849-5407 USA; 2 Biomedical Sciences Department, Florida State University, College of Medicine, 1115 West Call Street, Tallahassee, FL 32306 USA

**Keywords:** Drosophila, Sexual dimorphism, Insulin signaling, Gene expression, Sex bias, RNA-seq

## Abstract

**Background:**

The core functions of the insulin/insulin-like signaling and target of rapamycin (IIS/TOR) pathway are nutrient sensing, energy homeostasis, growth, and regulation of stress responses. This pathway is also known to interact directly and indirectly with the sex determination regulatory hierarchy. The IIS/TOR pathway plays a role in directing sexually dimorphic traits, including dimorphism of growth, metabolism, stress and behavior. Previous studies of sexually dimorphic gene expression in the adult head, which includes both nervous system and endocrine tissues, have revealed variation in sex-differential expression, depending in part on genotype and environment. To understand the degree to which the environmentally responsive insulin signaling pathway contributes to sexual dimorphism of gene expression, we examined the effect of perturbation of the pathway on gene expression in male and female Drosophila heads.

**Results:**

Our data reveal a large effect of insulin signaling on gene expression, with greater than 50% of genes examined changing expression. Males and females have a shared gene expression response to knock-down of InR function, with significant enrichment for pathways involved in metabolism. Perturbation of insulin signaling has a greater impact on gene expression in males, with more genes changing expression and with gene expression differences of larger magnitude. Primarily as a consequence of the response in males, we find that reduced insulin signaling results in a striking increase in sex-differential expression. This includes sex-differences in expression of immune, defense and stress response genes, genes involved in modulating reproductive behavior, genes linking insulin signaling and ageing, and in the insulin signaling pathway itself.

**Conclusions:**

Our results demonstrate that perturbation of insulin signaling results in thousands of genes displaying sex differences in expression that are not differentially expressed in control conditions. Thus, insulin signaling may play a role in variability of somatic, sex-differential expression. The finding that perturbation of the IIS/TOR pathway results in an altered landscape of sex-differential expression suggests a role of insulin signaling in the physiological underpinnings of trade-offs, sexual conflict and sex differences in expression variability.

**Electronic supplementary material:**

The online version of this article (10.1186/s12864-018-5308-3) contains supplementary material, which is available to authorized users.

## Background

Given the availability of sophisticated genetic tools, flexibility and low cost, Drosophila is an important model for understanding the role of the insulin/insulin-like signaling and target of rapamycin (IIS/TOR) pathway in aging, stress and metabolic disease [1–3]. IIS/TOR is highly conserved across metazoans, from worms to fruit flies to humans [4]. The extent of this conservation is illustrated by striking examples of functional conservation. For example, Drosophila protein extract can initiate insulin-like activity in mice [5, 6], and studies of chimeric human-fruit fly insulin receptors show that their cytoplasmic domains are functionally equivalent [7]. In addition, the suite of biological processes affected by insulin signaling is broadly similar in the animal systems studied thus far. This includes roles of IIS/TOR in metabolism, growth, nutrient sensing, stress, lifespan, and reproduction.

**Fig. 1:**
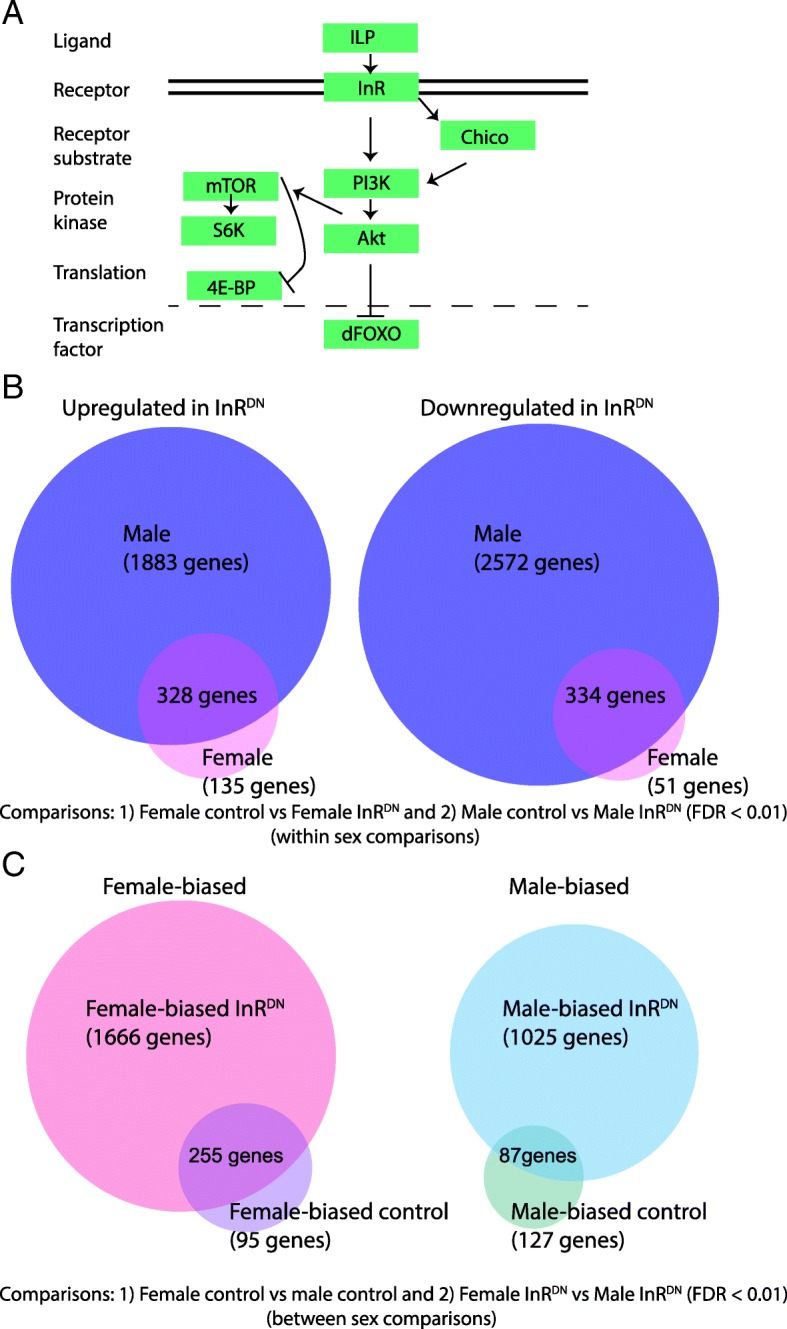
Sex differences in insulin signaling pathway responses. **a** Schematic of insulin signaling pathway. In Drosophila, insulin-like peptides (Ilps) bind the Insulin Receptor (InR), which is a transmembrane receptor. Upon Ilp binding, signal transduction through InR influences Foxo transcriptional activity and translation regulated by 4E-BP, among other changes. **b** Genes were identified that had significant differences in expression between the same sex control and InR^DN^-expressing flies, based on exon-level differential expression analyses. A gene is considered differentially expressed if at least one exon is differentially expressed. This set of comparisons is called “within sex comparisons”. The number of genes containing exons that change in both males and females, or only in one sex, is shown in the Venn diagram. Note that, If a gene is in the overlapping category (shows significant differential expression in multiple comparisons), this is based on exon-level analysis. **c** Genes were identified that had significant differences in expression between control males and females and between male and females expressing InR^DN^, based on exon-level differential expression analyses. A gene is considered differentially expressed if one exon is differentially expressed. This set of comparisons is called “between sex comparisons”. The number of genes with sex-biased expression in the control comparison, or in the InR^DN^-expressing comparison, or in both comparisons are shown in the Venn diagram. If a gene is in the overlapping category, this is based on exon-level analysis. For details regarding how these numbers were tabulated, see Additional file 5: Table S1A-B

The Drosophila extracellular components of the IIS/TOR pathway consist of insulin-like peptides, and the Insulin-like receptor (InR) (see Fig. 1a for Drosophila pathway). There are eight Drosophila insulin-like peptides (Ilps) that bind to the single, transmembrane, insulin-like receptor (reviewed in, [8–10]). Ilp activity is modulated by ImpL2 (encoded by *Ecdysone-inducible gene L2*), an insulin-like-growth-factor binding protein [11, 12], and at least one decoy receptor (encoded by *Secreted decoy of InR*) [13]. The Ilps modulate body size during development through IIS/TOR signaling. However, they are expressed in different subsets of cells and tissues, including brain insulin producing cells (IPCs), and head and abdominal fat body, with a variety of effects on physiology and behavior (reviewed in, [8–10, 14]). For example, Ilp2 is required for metabolism of carbohydrates, and Ilp3 and Ilp6 play roles in lipid metabolism [15, 16]. Complex interactions and feedback loops are also present. For instance upregulation of Ilp6 (expressed in glia, and the head and abdominal fat body), with consequent repression of Ilp2 and Ilp5 (expressed primarily in the brain IPCs), extends lifespan in Drosophila [17].

Following activation of InR and its intracellular substrate encoded by *chico*, a cascade of kinases and phosphatases directs activation or repression of downstream components of the pathway (see Fig. 1a). Ultimately, cell growth and proliferation are impacted by inhibiting activity of the transcription factor encoded by *forkhead box, sub-group O* (*foxo*), and by repressing negative regulators of the product of *target of rapamycin* (TOR) (reviewed in, [2, 18, 19]). Foxo is responsible for upregulation of stress responses and downregulation of genes and pathways that promote growth under high nutrient conditions [20–22], whereas TOR functions in regulating protein synthesis and cell growth [23]. The IIS/TOR pathway is also part of an extended, interacting network of hormone, and other, signaling pathways. Understanding the complete network and its myriad of functions is an area of active research [24].

A recurring theme in the biology of insulin signaling is regulation, or modulation, of sex differences throughout the life cycle. Insulin signaling is required for regulation of Drosophila sexual dimorphism during development, such as in body size [25], and in conditional expression of secondary sexual traits in other animals (reviewed in [26]). During Drosophila juvenile and adult stages insulin signaling modulates sexual maturity, behavior, and egg production [27–32]. This has led to the idea that hormone signaling, with insulin signaling as a prime example, plays an important role in sex determination in insects [33, 34].

Interrelated physiological processes such as aging and stress are also regulated by IIS/TOR signaling and this regulation is different in each sex [35–37]. Perturbation experiments in which one gene in the pathway (e.g., *foxo*, *chico*, or *InR*) is knocked-out, knocked-down, or overexpressed report significant dimorphism of aging and stress phenotypes [10, 38]. These experiments have shown substantial quantitative and qualitative differences between males and females in associated phenotypes. For example, mutations in *chico* can result in complex changes to lifespan, heat tolerance and resistance to oxidative stress, that differ between males and females [39].

A role for hormone signaling, and specifically the IIS/TOR pathway, in modulating sexually dimorphic traits is well established, but how IIS/TOR signaling impacts sex-differential expression has not been examined. This is important because modulation of sex-differential expression by this pathway may contribute to both genetic and environmental variation in natural populations, and differences in the degree of sex-differential expression found in existing studies. Further, mathematical and verbal models suggest that intralocus sexual conflict could be resolved by these types of regulatory mechanisms [40–43].

Our approach to delineating the role of insulin signaling in sex-differential expression was to examine the effects of perturbation of the IIS/TOR pathway on gene expression in adult Drosophila male and female heads. Drosophila adult head samples are enriched for nervous system (e.g. brain and sensory system) and endocrine (e.g. head fat body) tissues and have been the focus of our previous analyses of sex-differential expression [44–46]. Examining expression in head samples reduces complexity relative to whole adults, focuses the study on somatic cell-types that are present in both males and females, for which the sex determination hierarchy is well understood, and allows for identification of expression patterns associated with dimorphism of behavior, as well as metabolism and stress responses. This approach reveals which parts of the pathway, including downstream targets, respond differently between males and females. The overarching goal is to build a broad understanding of the regulatory response to IIS/TOR signaling in adult males and females, and to further understand how sex differences in gene expression arise.

## Results

To determine the impact of reduced insulin signaling during adult stages on gene expression, expression of a dominant-negative, insulin-like receptor (InR^DN^; [47]) was induced in virgin males and females four days after eclosion, for four days, using the drug-inducible GeneSwitch system [48, 49]. Given that both mammalian and Drosophila InR function as tetramers [5], with two α and two β subunits, expression of this dominant negative variant interferes with wild-type endogenous receptor activity [50]. The GeneSwitch system utilizes the yeast Gal4 transcription factor, with the heterologous progesterone receptor ligand binding domain fused to Gal4, rendering Gal4 inactive in the absence of ligand (see Additional file 1: Figure S1 for GeneSwitch schematic). The progesterone receptor agonist, RU486 (mifepristone), is added to the Drosophila food media to activate Gal4 transcription factor activity. Gal4 activates gene expression by binding UAS response element DNA. Thus, in the presence of RU486, expression of InR^DN^ is induced and insulin signaling is reduced [47, 50, 51]. In this study, GeneSwitch Gal4 was expressed ubiquitously, under the control of the actin promoter. Perturbation conditions resulted in 8-fold (males) and 14-fold (females) higher expression of the dominant negative *InR* transgene relative to endogenous InR expression under control conditions. This indicates that InR^DN^ is expressed at substantially higher levels than endogenous InR and is likely sufficient to reduce most/all signaling function ([52] see Additional file 1: Figure S1).

Gene expression was examined in adult males and females that were drug treated (RU486, dissolved in ethanol, added to food) and in age-matched controls (solvent for drug, ethanol, added to food). All animals used in this study were the same genotype (*UAS-InR*^*DN*^*/UAS-GFP; actin promoter-GeneSwitch*). mRNA from 50 heads per replicate (*n* = 4 replicates for each treatment) was obtained and used to make Illumina RNA-seq libraries, with 100 base, single-end sequence data collected. Drosophila adult head samples include multiple organs, tissues and cell-types, including the eyes, brain, muscle, and head fat body. The RNA-seq data was analyzed at the exon level, to gain insight into transcript-isoform expression differences (Additional file 2: Figure S2; see [53] for description), where a gene is considered significantly differentially expressed if at least one exon has differential expression (FDR < 0.01 for analyses presented; see Additional file 3 for FDR < 0.05 and < 0.0001).

Alternative transcripts and gene overlap can result in ambiguity with respect to the transcript identity of reads in RNA-seq. In our analysis reads are mapped to unique exonic regions, corresponding to single or overlapping exons from the same gene, whereas RNA-seq reads are not considered when they map with ambiguous gene identity [46, 53]. Overall, 39,462 exons corresponding to 8405 genes had a sufficient number of reads mapping to unambiguous gene regions to be included in the analysis. An ANOVA model was fit and pairwise contrasts used to identify significant differences in gene expression, following FDR correction.

### The response to reduced insulin signaling in males and females

To identify the genes that change expression in males and females due to InR^DN^ expression, we identified the genes within each sex that are induced or repressed, as compared to the same-sex control (hereafter called “within sex comparisons”; Fig. 1b; FDR < 0.01). The test for significant effects of reduced InR pathway signaling reveals that this perturbation has a large effect on gene expression (Fig. 1b; Additional file 4; Additional file 5: Table S1A). In this study, 4672 genes (55.6% of tested genes) showed a statistically significant response to perturbation in females, in males or in both sexes, and of these, 1289 (27.6% of tested genes) showed significant, two-fold or greater differences (Additional file 4).

There was a substantial difference between the sexes in the number of genes induced or repressed by the InR^DN^ signaling perturbation (Fig. 1b). There were 662 genes that showed significant responses in males and females, in the same direction. Genes that changed expression in both sexes were relatively equally split between those upregulated (*n* = 328) and those downregulated (*n* = 334). However, 1883 genes were upregulated, and 2572 genes downregulated, in InR^DN^-expressing males, as compared to control males. Whereas, in InR^DN^-expressing females, as compared to control females, only 135 genes were upregulated, and 51 genes downregulated. This shows that reduced insulin signaling has a larger impact on gene expression in males, relative to females.

**Fig. 2:**
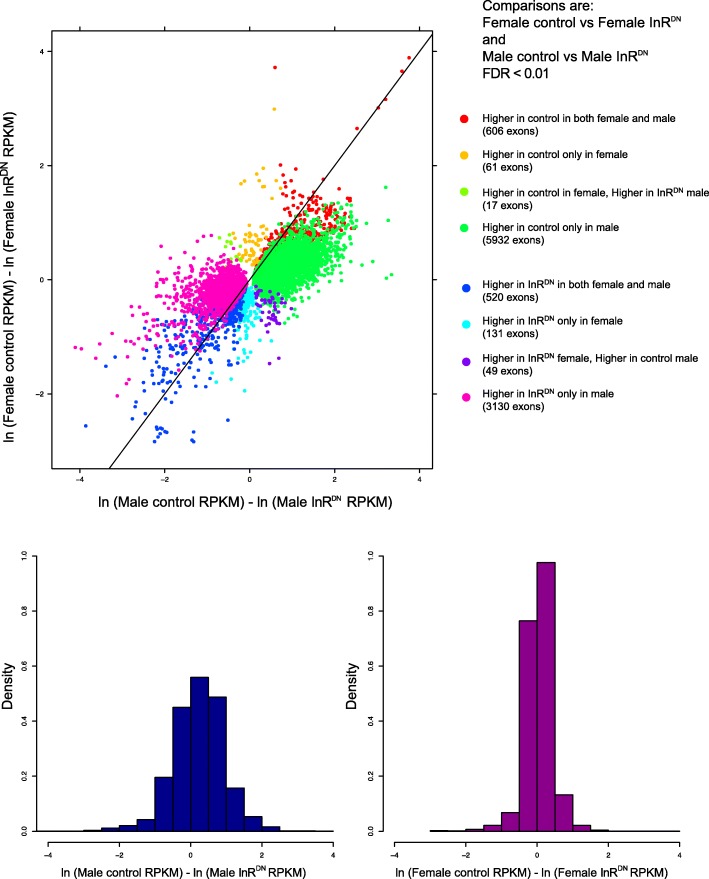
Exon-level estimates of expression differences within males and females. Exons with significant differences in expression between the control and InR^DN^-expressing flies (within sex comparisons; FDR < 0.01). The within sex comparisons are: 1) female control vs female InR^DN^-expressing flies and 2) male control vs male InR^DN^-expressing flies. The natural log of the fold change (ln-fold change) is plotted on the X-axis, for the male comparison, with positive values (right) indicating higher expression in control. The ln-fold change is plotted on the Y-axis, for the female comparison, with positive values (top) indicating higher expression in control. The histograms below show the distribution of the ln-fold change for the male (left; blue) and female (right; purple) comparisons. Colored dots indicate if the expression differences were significant in a given comparison. Black diagonal reference line shows line of equal change in expression in males and females, between control and InR^DN^ expression

Not only do males show a greater number of significant differences, the differences in expression are also greater in males (bottom panel, Fig. 2; Additional file 5: Table S1A). The largest increases and decreases in expression, on average, correspond to the exons showing significant differences in both males and females, in the same direction with respect to upregulation or downregulation (red and blue points, Fig. 2; for distributions within each category see Additional file 6: Figure S3). Significant differences which had opposing effects in males and females (light green and purple points, Fig. 2), showed a more reduced range of differences and were smaller on average compared to the other categories.

**Fig. 3:**
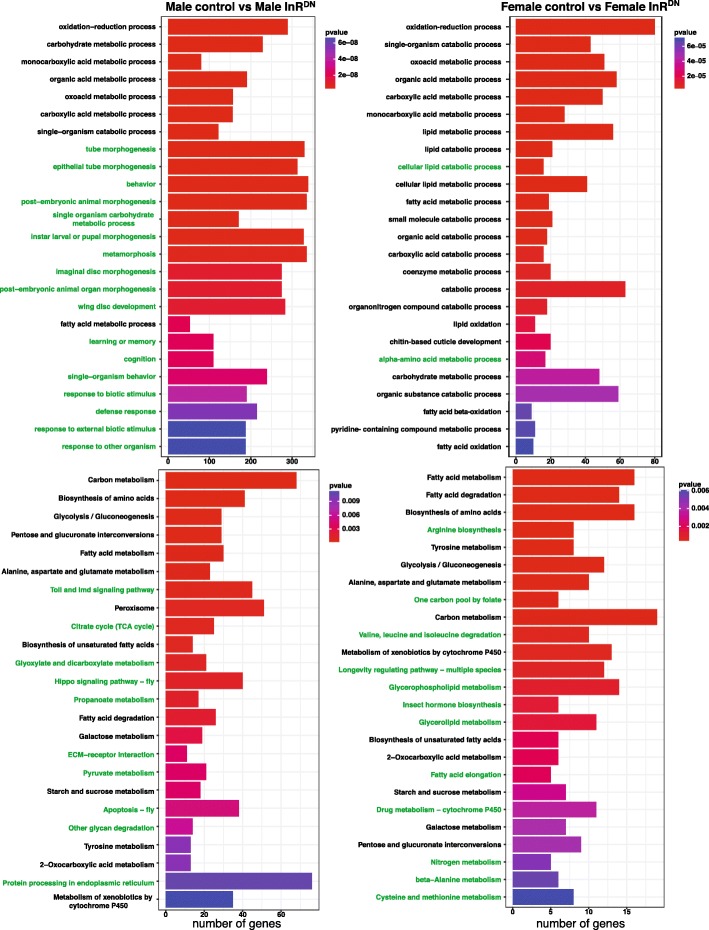
Reduced InR signaling impacts different pathways and functional gene groups in males and females. The GO Biological Process category (top) and KEGG pathway (bottom) with the most significant changes are shown for male (left) and female (right). The *p*-value is indicated by the red-purple scale, with red being most significant. The number of genes that were considered in the analyses is plotted on the X-axis. Pathways and groups that are uniquely significant in one sex are indicated by green font (FDR < 0.05)

To interpret the regulatory response to reduced insulin signaling with respect to pathway membership and gene function, gene set enrichment analysis was conducted for each within sex comparison (FDR < 0.05; [54, 55]). Kyoto Encyclopedia of Genes and Genomes (KEGG) pathways and Gene Ontology Biological Process (GO BP) categories were examined. Genes belonging to core metabolic pathways (KEGG) and those annotated as functioning in related biological processes (GO), for example ‘carbon metabolism’, ‘carbohydrate metabolism’, and ‘biosynthesis of amino acids’, were enriched in both sexes (Fig. 3). In some cases different, but functionally similar, categories were enriched in each sex. For example, the GO BP ‘aging’ category is enriched in the within male comparison’s genes and the KEGG ‘longevity regulated pathway’ is enriched in the within female comparison’s genes. More GO BP categories were uniquely enriched in the male comparison than in the female comparison (Fig. 3 and Additional file 7). Categories which were uniquely enriched among differentially expressed genes for the male within sex comparison included ‘immune response’, ‘defense response’, as well as ‘behavior’ and ‘learning or memory’. For females, the uniquely enriched categories included ‘insect hormone biosynthesis’, ‘triglyceride homeostasis’ and several ‘amino-acid metabolic process’ categories.

We also examined the responses of individual genes to perturbation of the InR pathway, in order to understand how the regulatory response is shared, or unique, with respect to the degree and direction of changes in gene expression. Genes that were downregulated two-fold in both sexes included those involved in insulin-like signaling and energy homeostasis, as expected. For example, 1) *adipokinetic hormone receptor* (*AkhR*), a glucagon-like receptor expressed in fat body and gustatory neurons [56], 2) *target of brain insulin* (*tobi*; [57]), an ɑ-glucosidase expressed in the gut and fat body that is known to be upregulated by both insulin-like and glucagon-like signaling, and 3) *insulin-like peptide 5* (*Ilp5*), expressed in brain IPCs, are all downregulated two-fold under perturbation conditions.

Other metabolic genes that are involved in lipid and carbohydrate homeostasis respond to the perturbation in both males and females. This includes down regulation of specific transcripts, for example of *trehalose hydrolysis enzyme*, *Treh*, which breaks down trehalose to glucose in the hemolymph and in glia [58–60] and overall down and upregulation, respectively, of lipid homeostasis genes expressed in the brain and fat body: *Lipid storage droplet protein 1* (*Lsd-1*), which activates triglyceride lipolysis and is downregulated under starvation, and *brummer* lipase (*bmm*) a triglyceride lipase, which is known to be upregulated under starvation [61]. These responses are expected, as perturbation of IIS/TOR signaling has been shown to produce effects similar to starvation [20, 23, 62]. The large effects on expression of well-characterized genes validate the overall approach taken here, and highlight and extend our insights regarding the overall shift in expression of metabolic genes that is observed under perturbation conditions in both sexes.

**Fig. 4:**
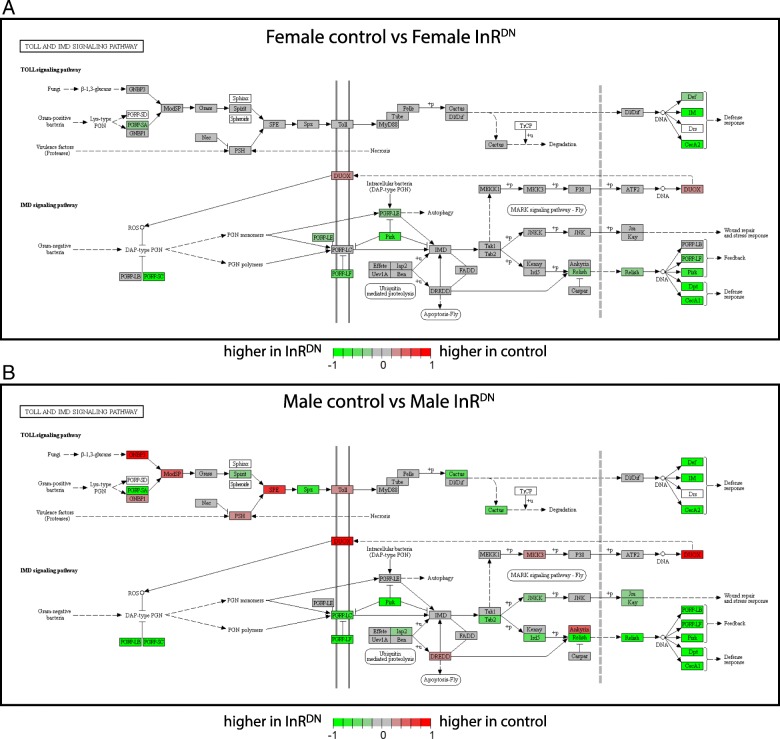
Expression differences in males and females in response to reduced insulin signaling for immune response genes. For genes in the Toll/Imd signaling pathway (KEGG: dme04624), the expression differences are indicated by color, with color gradations from − 1 or less, to 1 or greater, using KEGG graph and Pathview [101]. Female control vs female InR^DN^-expressing flies (**a**) and male control vs male InR^DN^-expressing flies (**b**) are shown. The data in this figure was not filtered by a statistical cut-off. Green indicates higher expression in InR^DN^-expressing flies and red indicates higher expression in control flies

However, thousands of genes showed sex-specific differential expression as a result of reduced insulin signaling (Additional file 4). Most of these showed male-specific changes in expression. Among the most striking pattern was male-specific upregulation or downregulation of immune response genes (Fig. 4; Additional file 7). This is consistent with the strong biological links between changes in metabolism and immune responsiveness [63–65]. While females shared some of these responses, both the number of genes and the magnitude of the effects were larger in males (Fig. 4). Immunity and defense genes with male-specific responses included those with a range of functions, for example genes encoding signal transduction proteins (e.g., *Relish*, *cactus*), pattern recognition proteins (e.g. *PGRP-LC*, *GNBP3*), and antimicrobial peptides (e.g. *Def*, *Dpt*). In other Drosophila transcriptomic studies of environmental perturbation, upregulation of the immune response was also detected, further highlighting the link between immune responsiveness and pathways that detect environmental signals [66, 67].

There were fewer genes with female-specific changes in expression under reduced insulin signaling. However, several genes known to be female-biased in Drosophila including *spinster* (*spin,* downregulated two-fold), *heimdall* (*hll,* upregulated two-fold), *Nidogen* (*Ndg,* upregulated two-fold), and *rolling stone* (*rost,* downregulated two-fold) show consistent, and female-only, regulatory responses to the perturbation. The *spin* locus encodes a putative permease with an MFS transporter family domain [68]. The gene was named based on the reduced mating propensity of female *spin* mutant strains, a phenotype also observed to be associated with reduced insulin signaling [69, 70]. More recently, *spin* has been shown to play a role in lipid metabolism, with some *spin* mutant genotypes producing extreme imbalances in lipid metabolites [68, 71]. *hll* also plays a role in lipid metabolism. Knock-down of *hll* reduces triglyceride levels and affects resistance to sleep deprivation, indicating a possible role for *hll* in energy homeostasis [72]. *Ndg* is a highly conserved component of basement membranes, but has not been studied extensively in Drosophila, and *rost* is involved in myoblast fusion [73, 74]. Both *Ndg* and *rost* are strongly female-biased in head tissues, and *rost* is a putative target of the Doublesex sex hierarchy transcription factor (see below for more on the sex hierarchy; [44, 46]).

### The landscape of sex-differential expression is altered by the perturbation

To determine the effects of reduced InR signaling on sex-differential expression, gene expression was compared: 1) between control male and females and 2) between InR^DN^-expressing male and females (hereafter, these comparisons will be called “between sex comparisons”). Of 8405 tested genes, 2980 (35.5%) are significantly, sex-differentially expressed in one or both conditions, of these 812 (27.2%) differed two-fold between males and females in transcript level in one or both of the comparisons (Additional file 4: Table S1B; Additional file 8).

There was a substantial shift in sex-differential expression, with a large gain of novel sex-differential expression occurring when insulin signaling was reduced (Fig. 1c). Considering the female-biased genes, 255 maintained female-bias in both conditions, while 1666 became female-biased in individuals expressing InR^DN^. For genes with male-biased expression, 87 were male-biased in both conditions, but 1025 showed a male-biased expression pattern in individuals expressing InR^DN^. There were an additional 95 genes that lost female-biased expression and 127 genes that lost male-biased expression when insulin signaling was reduced. Of these, a small number of genes showed changes in the direction of sex-differences from female- to male-biased or from male- to female-biased (32 genes).

**Fig. 5:**
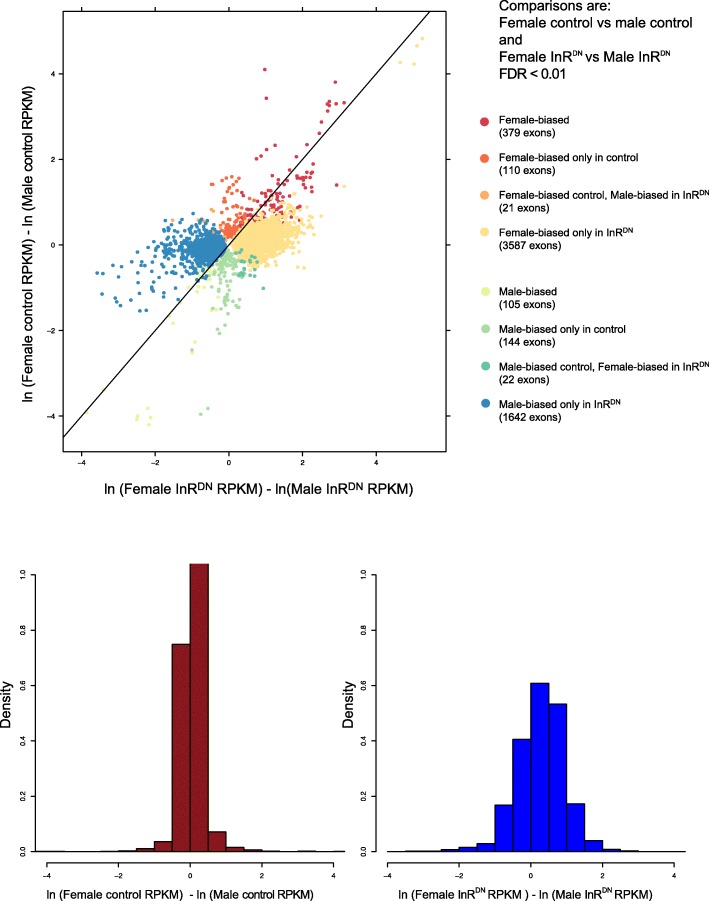
Exon-level estimates of sex-differential expression between control and InR^DN^-expressing conditions. Exons with significant differences in expression between the sexes (between sex comparisons; FDR < 0.01). The between sex comparisons are: 1) female vs male control and 2) female vs male InR^DN^-expressing flies. The ln-fold change is plotted on the X-axis, for the InR^DN^-expressing comparison, with positive values (right) indicating higher expression in females. The ln-fold change is plotted on the Y-axis, for the control comparison, with positive values (top) indicating higher expression in females. The histograms below show the distribution of the ln-fold change for the control (left; brown) and InR^DN^-expressing flies (right; blue) comparisons. Colored dots indicate if the expression differences were significant in a given comparison. Black diagonal reference line shows line of equal sex differences in expression between control and InR^DN^ expression

There were more, and larger, differences between males and females on average with InR^DN^ expression (bottom panel, Fig. 5). The perturbation conditions were primarily associated with gain of sex-differential expression, largely due to male-specific downregulation and upregulation (green and pink points, Fig. 2). However, differences in expression between sexes were greater, on average, for exons that maintained the same pattern of sex-differential expression in both control and treatment conditions than they were for the other categories (red and chartreuse points, Fig. 5; for distributions in each category see Additional file 9: Figure S4). Sex-differences which changed in the direction of bias across conditions were smaller, on average, than those observed for the other categories (orange and dark-green points, Fig. 5).

To determine if the observed changes in sex-differential expression, due to InR^DN^ expression, show functional differences, we examined enrichments for GO BP and KEGG categories. The top GO BP and KEGG pathways showing enrichment among genes that were sex-differentially expressed under perturbation conditions included ‘Toll and Imd signaling’, ‘carbon metabolism’, ‘biosynthesis of amino acids’, ‘fatty acid metabolism’ and ‘insect hormone biosynthesis’ (Additional file 10: Figure S5).

To gain insight into how reduced insulin signaling may be modulating sex-differential expression, we examined how sex-differential expression changed in individual genes. As expected, many genes known to show male- or female-biased expression in heads, for example *Yp3*, *fit*, and *Ndg* in females and *sxe1*, *roX1* and *roX2* in males, maintained sex-differential expression in both conditions, which also provides validation of the approach (For examples see [44–46]). Other *yolk protein* genes showed large and female-biased expression level in both conditions, but the corresponding exons were not always significant at FDR < 0.01 in control conditions. We note that the FDR was < 0.05 for all *yolk protein* genes in control conditions (Additional file 3).

However, in the majority of cases, sex-differential expression was detected in response to expression of InR^DN^, for genes (or exons within genes) that were not significantly different between males and females under the control conditions. In some cases these shifts were quantitative, and expression was altered in both sexes with reduced insulin signaling, but to differing degrees. For example *Juvenile hormone epoxide hydrolase 1* and *2* (*Jheh1* and *Jheh2)*, which degrade juvenile hormone and function in resistance to oxidative stress [75, 76], are reduced in both sexes when InR^DN^ is expressed. However, the reduction in males is greater than in females leading to female-biased gene expression (Additional file 11: Figure S6). Resistance to oxidative stress has been shown to be sexually dimorphic in Drosophila, but it is not clear if *Jheh1* and *Jheh2* play roles in these differences [77, 78]. In other cases changes appear to be qualitative, and a regulatory response to reduced insulin signaling was observed in one sex and not in the other. For example, from the perspective of sex-differential expression, female-only down regulation in the expression of *spin* (nine of ten exons) produces a shift to male-biased expression (Additional file 11: Figure S6). The genes *sex-specific enzyme 1* and *2* (*sxe1* and *sxe2),* were identified in previous studies with male-biased expression in head fat body, where both genes are regulated by the sex determination hierarchy and are rhythmically expressed [44, 46, 79–81]. *sxe1*, a cytochrome with a role in male mating success [80], shows reduced expression in the treatment condition, but the male-biased expression pattern does not change. In contrast, the lipase *sxe2* is male-biased in the control conditions, but loses male-bias in the treatment condition and is instead expressed at similar, lower levels in both females and males.

One possible explanation for the effects of the perturbation on sex-differential expression is changes in expression of the terminal transcription factors in the sex determination hierarchy, encoded by *doublesex* (*dsx*) and *fruitess* (*fru*). The sex hierarchy consists of an alternative pre-mRNA splicing cascade, responsive to the number of X chromosomes, that results in production of sex-specific transcription factors: Dsx^M^, Dsx^F^, and Fru^M^. Dsx isoforms specify nearly all somatic sex differences outside the nervous system, with additional functions in small subsets of neurons, whereas Fru^M^ is responsible for the potential for male reproductive behaviors through expression in 2–5% of the nervous system (reviewed in, [82–84]). Previous studies showed that genes in the sex determination hierarchy can regulate InR expression [25, 85]. We asked does InR regulate *dsx* and/or *fru*, thus contributing to the sex differences in response to expression of InR^DN^? We found that *dsx* is consistently downregulated under perturbation conditions in both sexes (Additional file 11: Figure S6). Some exons of *fru* are also affected, however the pattern is not consistent across the gene, possibly indicating isoform-specific effects (Additional file 11: Figure S6). The sex hierarchy genes show quantitative changes in expression, but are still produced, and most genes with expected sex-differential expression maintain male- or female-bias. This suggests that most of the sex differences we observe as a result of InR^DN^ expression are not exclusively due to alterations in the sex hierarchy terminal transcription factors.

### Sex differences in the response to insulin signaling within the IIS/TOR pathway

**Fig. 6:**
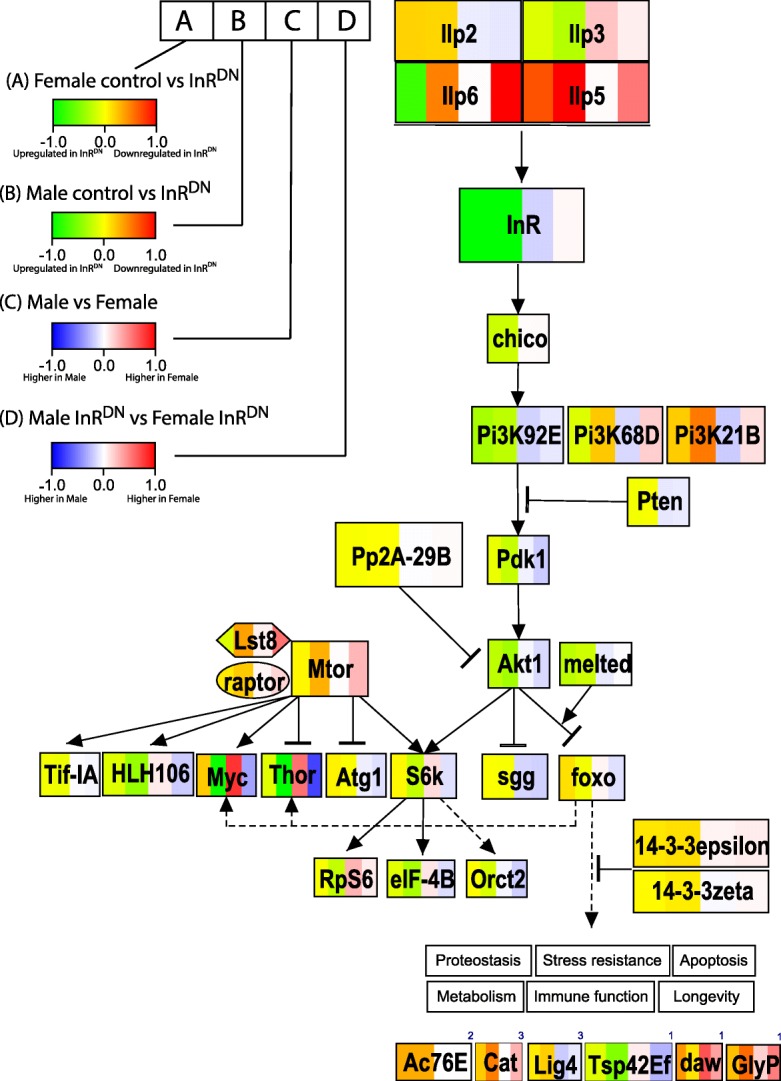
Expression differences within the IIS/TOR pathway for the four comparison types. For genes in the IIS/TOR pathway, the expression difference are indicated by color, with color gradations from − 1 or less, to 1 or greater, using PathVisioRPC [102]. Each gene is broken into four boxes (left to right), with the expression differences for the four comparisons shown (see legend on left). The data in this figure was not filtered by a statistical cut-off. On the bottom, the change in expression for six genes downstream of IIS/TOR is shown, with the citation indicated above the gene (1) [89] (2) [21] and (3) [103]. Note that the expression differences are calculated as the difference between mean expression estimates, as ln-RPKM, and differences are equivalent to the ln-fold change

There are also large and significant changes in expression of individual genes in the IIS/TOR pathway itself, with each gene showing unique responses in the four comparisons that we focused on in this study (Fig. 6). In this study reduced insulin signaling had different effects on each of the detected InR ligands (Ilp2, Ilp3, Ilp5 and Ilp6; Fig. 6 and Additional file 11: Figure S6). For example, *Ilp5* expression is repressed in both sexes, while *Ilp6* shows opposing patterns of expression in males and females. *Ilp6* is not sex-biased under control conditions (indicated by third white box; Fig. 6), but is upregulated in females and downregulated in males in response to expression of InR^DN^ (fourth red box; Fig. 6). The sex difference in the response of *Ilp6* results in greater than two-fold female biased expression under perturbation conditions. We also found that expression of InR^DN^ increased expression of the endogenous InR gene in both males and females, but the increase is greater in males than in females (Additional file 1: Figure S1). These changes in expression of *Ilp* and *InR* genes are likely to result from complex feedback loops that modulate IIS/TOR signaling [86]. Sex-dimorphism in expression of these ligands could therefore result from sex differences in known feedback mechanisms (e.g. by Foxo; [87, 88]) or from interactions involving sex-differences in the effect of the perturbation on downstream IIS/TOR pathway genes.

There are also changes in sex-differential expression of targets of the Foxo transcription factor (bottom of Fig. 6). *Tsp42Ef*, *daw* and *glyP* are Foxo targets associated with lifespan that show different expression responses to reduced insulin signaling in males and females [89]. Again, each of these genes showed a unique response to the four comparisons we focused on in these studies. Sex-differences in expression of these gene could indicate either *cis*/*trans* regulatory differences between males and females, for example in the overall activity of Foxo (as suggested in [38]) or in interactions between Foxo and its binding site for specific genes.

Strikingly, two IIS/TOR pathway cell growth regulation genes, *4E-BP* (*Thor*) and *Myc*, show shifts in the direction of sex-differential expression from female-biased patterns of expression under control conditions (third box for each gene is red, Fig. 6), to male-biased patterns under perturbation conditions (fourth box for each gene is blue, Fig. 6). The gain of a male-biased expression pattern results from male-specific upregulation. Interestingly, male-specific upregulation of *4E-BP* and *Myc* under perturbation conditions could be associated with the male-specific enrichment of innate immune response genes, among those genes showing a regulatory response to the perturbation. *4E-BP* is upregulated in response to infection or wounding and mutations in *4E-BP* result in a compromised immune response [65]. *Myc* is upregulated in response to dietary restriction and overexpression of *Myc* results in increased survivorship following infection [64]. Foxo is also known to regulate expression of anti-microbial peptides (AMPs), independently of the Toll or IMD pathways [90]. Thus, if there is sex dimorphism in activity of Foxo this could contribute to the differences we observe in expression of Foxo targets, including AMPs. We note that while sex differences in the immune response have been observed in Drosophila [91, 92], to the best of our knowledge there have been no studies which examine the potential role of 4E-BP, Myc or Foxo in these differences in Drosophila. The existing studies were done in only a single sex or in mixed sex groups.

## Discussion

In this study we show that males and females have different gene expression responses to perturbation of the insulin signaling pathway in adult heads, which include a diverse set of somatic cell types, tissues and organs with a broad range of functions, spanning from nutrient sensing to reproductive behavior. We find approximately an order of magnitude more genes changing expression in males, as compared to females (Fig. 1). The difference in responses between the sexes results in an increase in the number of genes sex-differentially expressed under perturbation conditions. These differences do not appear to be driven exclusively by the sex hierarchy terminal transcription factors in the adult. Rather, we find that there are quantitative changes in expression of *dsx* and *fru* genes encoding the terminal transcription factors when insulin signaling is reduced (Additional file 11: Figure S6), but known, well-validated, targets maintain the expected dimorphic expression patterns in both control and perturbation conditions.

A direct examination of the insulin signaling pathway showed that many genes responded sex-differentially to expression of InR^DN^ (see for example *Myc*, Fig. 6). The clear differences in male and female responses to reduced insulin signaling within the IIS/TOR pathway itself could explain some sex differences in physiology. For example, in the link between insulin signaling and aging or stress responses [35, 38, 93, 94]. Here, we find sex-differences both in expression of extracellular ligands (upstream of InR) and in downstream targets of the terminal transcription factor Foxo. These changes show that males and females are likely to differ both in gene expression changes involved in the initial response to insulin signaling reduction and in feedback loops that may buffer these types of perturbations (e.g. in *Ilp* expression).

Thus, we posit that for some genes that gain sex-differential expression due to insulin signaling perturbation, there is an interaction between the sex hierarchy and insulin signaling pathways. For example, *dsx* expression in adult fat body actively regulates sex differences in expression of *yolk protein* genes, which are also downregulated in response to perturbation of insulin signaling. Additionally, the sex hierarchy establishes morphological and physiological sexual dimorphism during development, and these differences could drive the sex-differences in gene expression observed in adults. For example, the sex hierarchy establishes differences in the somatic gonadal tissues, and signaling between the gonad and head fat body is likely to be different in males and females under insulin signaling perturbation. In fact, we find large changes in expression of *Ilps* that differ between males and females. Ilps are known to be involved in signaling between the gonad and head, with ablation of the gonad resulting in very large changes in expression of these genes [95].

Our results show that adult perturbation of IIS/TOR signaling has a larger impact in males on gene expression. This is distinct from studies that have examined the loss of sexual dimorphism at the phenotypic level with reduced or absent insulin signaling, for example the loss of sexual size dimorphism or dimorphic locomotor activity in adults [25, 30, 96]. Sexual dimorphism in body size, with larger females, was shown to partially be due to increased Ilp expression in female fat body tissue, as a result of the female-specific product of the sex hierarchy gene *tra*. This suggests that IIS/TOR has more of an impact in females than males during development [25]*.* Furthermore, *InR* mutant males and females do not have dimorphic body size, with both sexes showing a large reduction in mass [96]. In our study we do find that a small subset of genes require insulin signaling to maintain dimorphic expression in adult head tissues, but the majority gain dimorphism with reduced insulin signaling. Our results, together with those examining dimorphism in stress and aging in adult stages, demonstrate the importance of further studies aimed at understanding how these differences at the level of gene expression map to differences in phenotype in adult Drosophila, as well as examining sexual dimorphism of IIS/TOR function in different contexts.

Sex-differential expression has been examined in multiple studies in Drosophila head tissues and there has been variation in the genes with sex-differential expression [44, 45, 97]. In our previous work on gene expression variation downstream of Dsx, we found substantial effects of strain genetic background, indicating genetic or gene by environment variation affecting sex-differential expression in head tissues [46]. Our study showed that not all genes with the potential to be sex-differentially expressed are detectably sex-differentially expressed, in a particular genotype under laboratory conditions. This is despite the fact that these are likely bona fide targets, based on multiple, genome-scale studies [98, 99]. This suggests that there are modifiers of sex-differential expression acting in the adult, such that whether or how Dsx is affecting expression is dependent on either internal or external conditions. The results of this study highlight how some of the variation in sex-differential expression could occur, with insulin signaling generating sex-differential expression. Thus, natural variation in genes in the insulin signaling pathway or variation in any aspect of feeding/nutrient acquisition has the potential to impact sex-differential expression in the adult. These results point to a need for understanding the complex interactions between genetic variation, environmental variation and their interactions to understand sex-differential expression.

## Conclusions

The insulin signaling pathway is central to the physiological processes that underlie energy homeostasis and stress responses, redirecting investment of organismal resources from reproduction into somatic maintenance and survival, depending on environmental factors that affect dietary energy intake. These data support a common metabolic response in males and females, in terms of genes and pathways, but with many of the genes/pathways in common displaying quantitative differences in degree of expression changes. We also uncover genes/pathways that change only in one of the sexes, with both changes in degree and presence/absence differences driving sex-differential expression under perturbation conditions. These results contribute to our understanding of how sex differences in gene expression arise, as well as the mechanisms driving variation in dimorphic expression. This study reveals clear links between insulin signaling and regulatory responses expected to affect aging, lifespan, immunity, stress and behavioral differences between males and females. Thus, to fully understand sexual dimorphism in any of these processes, it will be important to understand their mechanistic interactions, within each sex, as well as their evolutionary origins.

## Methods

### Fly husbandry, tissue collection and library preparation

Flies were raised on standard cornmeal food medium (33 L H2O, 237 g Agar, 825 g dried deactivated yeast, 1560 g cornmeal, 3300 g dextrose, 52.5 g 9 Tegosept in 270 ml 95% ethanol and 60 ml Propionic acid). The incubator conditions are 25 °C on a 12 h light: 12 h dark cycle. The genotype of the F_1_ flies analyzed is *yw; P{UAS-InRDK*^*1409A*^*}/P{w + [+mC] = UAS-GFP.S65 T}Myo31DF[DT2];P{w + [+mC] = Act5C-**GAL4.Switch.PR**}3*, generated from a cross between parental virgin female *yw; P{UAS-InRDK*^*1409A*^*}* and male *yw/Y;P{w + [+mC] = UAS-GFP.S65 T}Myo31DF[DT2];*

*P{w + [+mC] = Act5C-**GAL4.Switch.PR**}3/TM6B*. Drosophila strains were obtained from the Bloomington Drosophila Stock Center*.*

Virgin male and female F_1_ flies are collected soon after eclosion and aged for 3 days. On day four the flies are transferred to food medium with either 200 μM RU486 (80% ethanol is the solvent) or with the same volume of 80% ethanol for four days. Flies are then snap frozen in liquid nitrogen and stored at − 80 °C. Adult heads were separated from the body by mechanical tapping in the cryovial and then separated while frozen on a piece of plexiglass cooled on dry ice. Frozen heads were immediately transferred to Trizol reagent. Total RNA was extracted using the Trizol reagent protocol (Thermo Fisher) and was DNAse treated with Turbo DNAse and purified (Zymo).

For each experimental condition, libraries were prepared with *n* = 4 independent biological replicates, with 50 heads per replicate. RNA-seq libraries were generated using 2 μg total RNA. The NEBNext Ultra RNA Library Prep Kit for Illumina, with the polyA purification module (New England Biolabs Inc. E7530L), was used for Illumina library generation. Pooled, barcoded, libraries were sequenced on an Illumina HiSeq 2500 (100 BP, single end).

### Data processing and analysis

Duplicate reads were removed prior to mapping and distinct (non duplicate) reads were mapped to the FlyBase Release 5 genome using bwa v0.7.15 (bwa mem -M), retaining uniquely mapped reads for analysis. Expression was estimated as the normalized read count (ln-RPKM) at the exon level (including for regions of exon overlap, see [46, 53]) and following FB5.51 annotation. Because low coverage can significantly bias results, total read counts and average coverage were considered in addition to other quality metrics after initial mapping and alignment. Two samples were removed from consideration due to low coverage. To account for any differences in variance across groups, such as males and females, an analytical approach was taken that is robust to heterogeneity of variance.

Exons were considered detected if the average per nucleotide coverage (APN) was greater than five in two or more of the replicates for a given sample in at least one of the four sample types. Exons which were not detected according to these criteria and all ambiguous regions that corresponded to multiple different gene annotations, were excluded from the analysis. There were 33,230 single exons and 6232 regions of exon overlap. In total, 39,462 exons were retained in the statistical analysis (8405 genes).

A linear model was fit and tests for differential expression were performed for each comparison as pairwise contrasts, for each exon, using Kenward-Roger F-tests [100]. The within sex comparisons are *1*) female control to female expressing InR^DN^, *2*) male control to male expressing InR^DN^, *3*) female control to male control, and *4*) female expressing InR^DN^ to male expressing InR^DN^. Significance was considered at FDR < 0.0001, FDR < 0.01 and FDR < 0.05 [54]*.* All figures and tables report the results with FDR < 0.01. A fold-change greater than 2 was additionally considered in some cases. All analytical results are reported in Additional files 3, 4 and 8.

Gene set enrichment analysis (GSEA) of Gene Ontology (GO) and Kyoto Encyclopedia of Genes and Genomes (KEGG) pathways were conducted in R, clusterProfiler package, using a hypergeometric test for enrichment [55]. Enrichment was tested for each annotation type (GO Biological Process or KEGG pathway) and each comparison type (1–4 above) separately. Only genes with the relevant annotation were used in the background set for these tests. Enrichments were considered significant at FDR < 0.05 [54]*.*

## Additional files

Additional file 1: **Figure S1**: GeneSwitch system and genetic cross used in this study. (PDF 741 kb)

Additional file 2: **Figure S2**: Illustration of exonic regions and nomenclature. (PDF 517 kb)

Additional file 3: Exon Level Results. (XLSX 30719 kb)

Additional file 4: Within Sex Comparison Gene Level Categories. (XLSX 326 kb)

Additional file 5: **Table S1**: A and B (Word document. docx). (DOCX 28 kb)

Additional file 6: **Figure S3**: Within sex comparison: Histograms. (PDF 618 kb)

Additional file 7: KEGG and GO Biological Process Enrichments. (XLSX 106 kb)

Additional file 8: Between Sex Comparison Gene Level Categories. (XLSX 212 kb)

Additional file 9: **Figure S4**: Between sex comparison: Histograms. (PDF 404 kb)

Additional file 10: **Figure S5**: GO and KEGG enrichments. (PDF 1064 kb)

Additional file 11: **Figure S6**: Bar charts showing expression for individual exons for genes. (PDF 547 kb)

## Abbreviations

*AkhR*: *Adipokinetic hormone receptor*
*bmm*: *Brummer* lipase
BP: Biological Process
*dsx*: *Doublesex*
*Foxo*: *Forkhead box, sub-group O*
*fru*: *Fruitess*
GO: Gene Ontology
*hll*: *Heimdall*
IIS: Insulin/insulin-like signaling
Ilps: Insulin-like peptides
*ImpL2*: *Ecdysone-inducible gene L2*
*InR*: *Insulin-like receptor*
InR^DN^: Dominant-negative, insulin-like receptor transgene
KEGG: Kyoto Encyclopedia of Genes and Genomes pathways
*Lsd-1*: *Lipid storage droplet protein 1*
*Ndg*: *Nidogen*
*rost*: *Rolling stone*
*roX1*: *RNA on the X 1*
*roX2*: *RNA on the X 2*
*spin*: *Spinster*
*sxe1*: *Sex-specific enzyme 1*
*sxe2*: *Sex-specific enzyme 2*
*tobi*: *Target of brain insulin*
TOR: *Target of rapamycin*
*Treh*: *Trehalose hydrolysis enzyme*
