# Supplementary material for: Perturbation of IIS/TOR signaling alters the landscape of sex-differential gene expression in Drosophila
Source: BMC Genomics. 2018 Dec 10;19:893. doi: 10.1186/s12864-018-5308-3 (PMC6288939; doi:10.1186/s12864-018-5308-3)

Figure S1:

GeneSwitch system and genetic cross used in this study. *GeneSwitch* (*GS*) encodes a progesterone receptor ligand binding domain::Gal4 DNA binding domain chimeric protein. GS can not bind DNA in the absence of ligand (RU486). Ligand bound GS activates gene expression of InR^{DN} in adults upon RU486 addition to the food medium. Male and female animals with actin-GS and UAS-InR^{DN} are produce from a single cross.

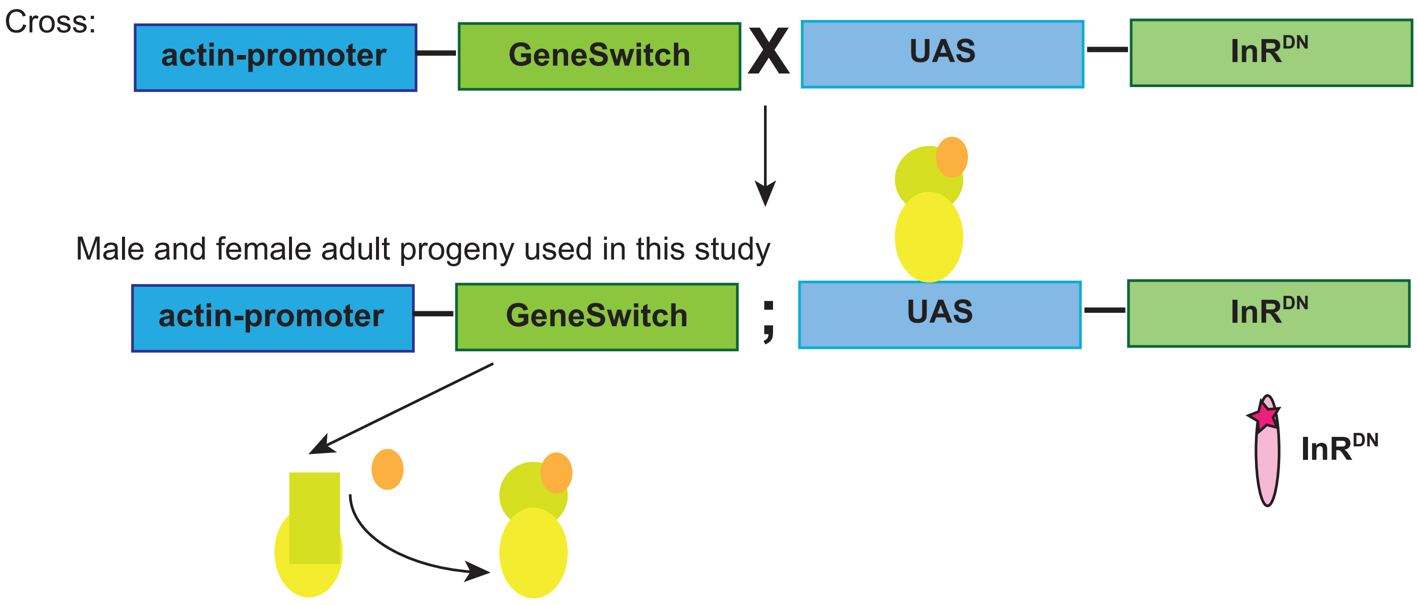

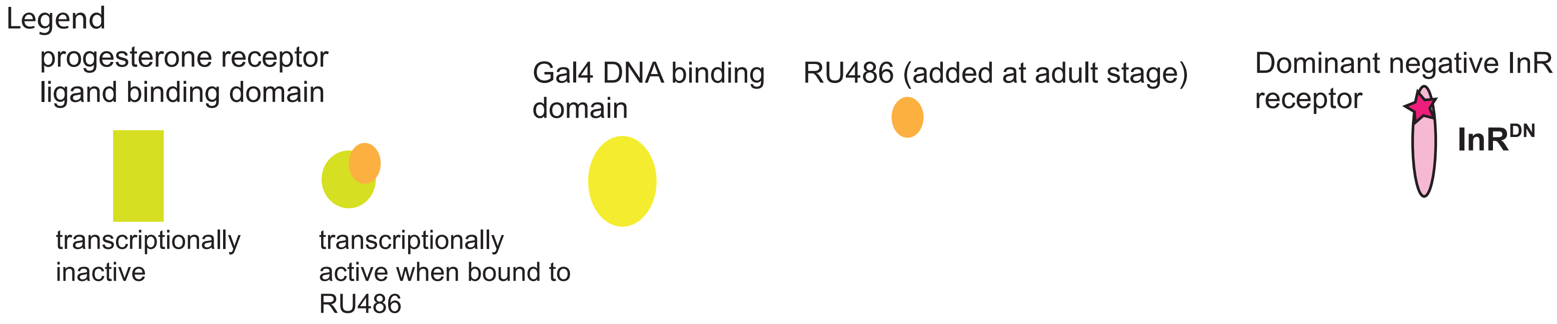

Overexpression of InR dominant negative transgene. Bar chart shows mean estimated expression (RPKM) for all detected exons of InR. Exons1-3 are expressed only from the endogenous InR gene and exons 4-13 are expressed from both the endogenous gene and the dominant negative transgene. Error bars represent one standard deviation. There were 13 exons with detected expression in the study. Exon 1 is the most 5' exon for isoform RA. The FlyBase exon name is in parentheses (FB5.51 annotation). The expression in females is shown in blue (control) and orange (drug treated).
The expression in males is shown in grey (control) and yellow (drug treated).

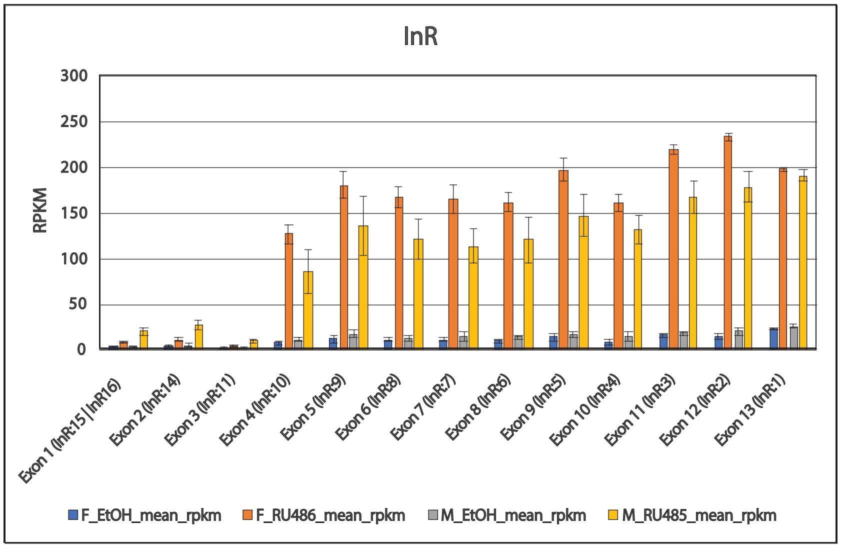

Supplement: Supplementary file 1 — Figure S1: GeneSwitch system and genetic cross used in this study. (PDF 741 kb) [file 12864_2018_5308_MOESM1_ESM.pdf]
