# Supplementary material for: Perturbation of IIS/TOR signaling alters the landscape of sex-differential gene expression in Drosophila
Source: BMC Genomics. 2018 Dec 10;19:893. doi: 10.1186/s12864-018-5308-3 (PMC6288939; doi:10.1186/s12864-018-5308-3)

Figure S2: Illustration of types of exonic regions and nomenclature.

Exonic regions were classified as belonging to either a single exon (S#_SI), or mutiple overlapping exons, F#_SI.

An F designation indicates that there are overlapping exons from either the same gene or different genes. In this study only exonic regions from single genes are considered, due to the ambiguity of assigning reads to regions that are multi-genic.

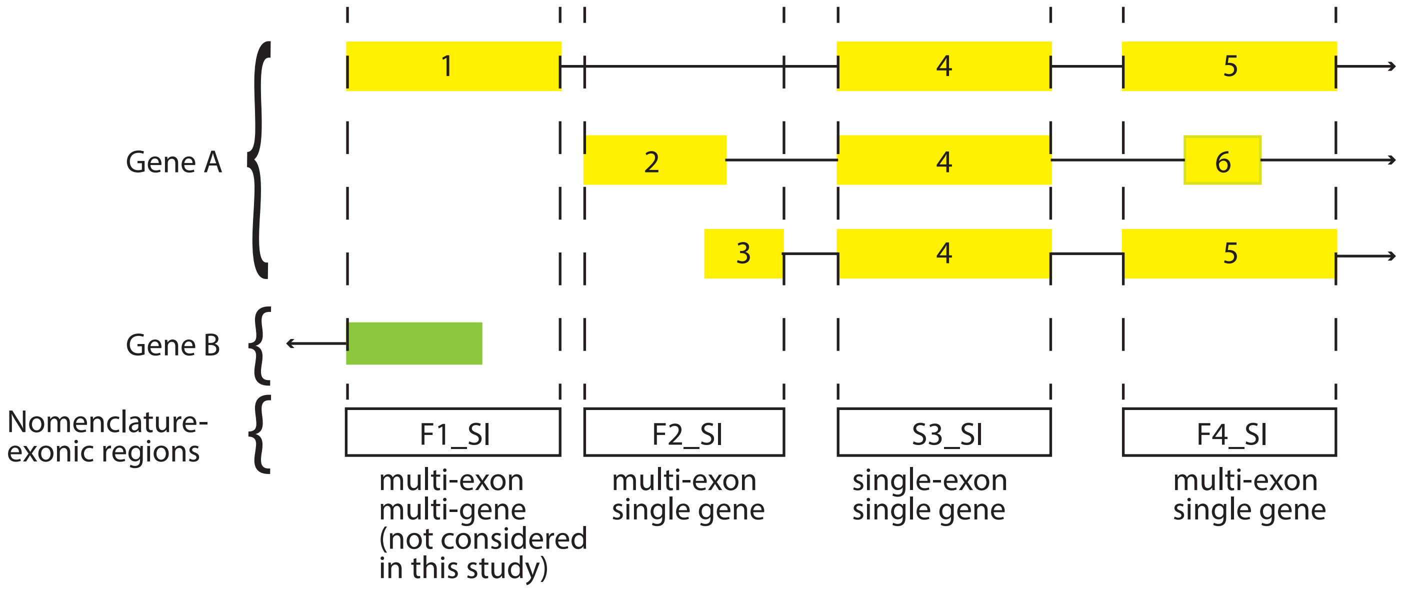

Supplement: Supplementary file 2 — Figure S2: Illustration of exonic regions and nomenclature. (PDF 517 kb) [file 12864_2018_5308_MOESM2_ESM.pdf]
