# Supplementary material for: Perturbation of IIS/TOR signaling alters the landscape of sex-differential gene expression in Drosophila
Source: BMC Genomics. 2018 Dec 10;19:893. doi: 10.1186/s12864-018-5308-3 (PMC6288939; doi:10.1186/s12864-018-5308-3)
Supplement: Supplementary file 5 — Table S1: A and B (Word document. docx). (DOCX 28 kb) [file 12864_2018_5308_MOESM5_ESM.docx]

**Table S1A-B**: **Summary of differentially expressed genes for each statistical comparison.** A: Genes were identified that had significant differences in expression between the same sex control and InR^DN^-expressing flies, based on exon-level differential expression analyses.  This set of comparisons is called “within sex comparisons”. The within sex comparisons are: 1) Female control vs Female InR^DN^ and 2) Male control vs Male InR^DN^ (FDR < 0.01). B: Genes were identified that had significant differences in expression between the sexes in control and InR^DN^-expressing comparisons. This set of comparisons is called “between sex comparisons”. The between sex comparisons are: 1) Female control vs male control and 2) Female InR^DN^ vs Male InR^DN^ (FDR < 0.01). For A and B: The number of genes in a category is based on the differential exon (DE) expression analysis. A gene is considered differentially expressed if at least one exon is differentially expressed. The percent of total Genes (Exons) is out of the number of genes, or their corresponding exons, included in the analysis (n=8405 genes and n=39,462 exons). In addition, the number of genes that had exons with two-fold or greater differences in expression were tabulated (ln-fold change greater than 0.69 or less than -0.69). Categories are based on exon level assignments. In some cases the exons of a gene may be placed in different categories. For this reason, genes can belong to multiple categories on the basis of these exon level assignments. If an exon had opposing effects in each condition (rows four and eight of each table), for example ‘Expression is higher in control in females, higher in InR^DN^ in males’, it was included in both corresponding counts, for example ‘Downregulated in InR^DN^ in females’ and ‘Upregulated in InR^DN^ in males’, in **Figure 1B** and **Figure 1C**.

**Table S1A: Differentially expressed genes for within sex comparisons.**

| **DE category (FDR < 0.01)** | **Genes (Exons)** | **%Total** | **Two-Fold** | **%Two-fold** |
| --- | --- | --- | --- | --- |
| Expression is higher in control in both sexes | 334 (606) | 3.97 (1.54) | 108 (218) | 1.28 (0.55) |
| Expression is higher in control in females | 40 (61) | 0.48 (0.15) | 18 (29) | 0.21 (0.07) |
| Expression is higher in control in males | 2,541 (5,932) | 30.23 (15.03) | 800 (2,007) | 9.52 (5.09) |
| Expression is higher in control in females, higher in InR^DN^ in males | 11 (17) | 0.13 (0.04) | 1 (1) | 0.01 (0.00) |
| Expression is higher in InR^DN^ in females | 104 (131) | 1.24 (0.33) | 25 (32) | 0.30 (0.08) |
| Expression is higher in InR^DN^ in males | 1,872 (3,130) | 22.27 (7.93) | 347 (576) | 4.13 (1.46) |
| Expression is higher in InR^DN^ in both sexes | 328 (520) | 3.90 (1.32) | 79 (167) | 0.94 (0.42) |
| Expression is higher in InR^DN^ in Females, but higher in control in males | 31 (49) | 0.37 (0.12) | 1 (1) | 0.01 (0.00) |

Note, in some cases the different exons of a gene corresponded to different categories. In these cases genes were flagged as belonging to multiple categories. For the within sex comparisons n = 4,099 genes were included in only one category at FDR < 0.01 and n = 573 genes were placed in more than one category at FDR < 0.01. For ln-fold change > 0.69 or < -0.69 and FDR < 0.01, n = 1,199 genes were included in only one category at FDR < 0.01 and n = 90 genes were placed in more than one category at FDR < 0.01.

**Table S1B**: **Differentially expressed genes for between sex comparisons.**

| **DE category (FDR < 0.01)** | **Genes (Exons)** | **%Total** | **Two-Fold** | **%Two-Fold** |  |
| --- | --- | --- | --- | --- | --- |
| Female-biased | 255 (379) | 3.03 (0.96) | 43 (86) | 0.51 (0.22) |  |
| Female-biased only in control comparison | 79 (110) | 0.94 (0.28) | 15 (27) | 0.18 (0.07) |  |
| Female-biased only in InR^DN^ comparison | 1,650 (3,587) | 19.63 (9.09) | 537 (1,282) | 6.39 (3.25) |  |
| Female-biased in control comparison,  Male-biased in InR^DN^ comparison | 16 (21) | 0.19 (0.05) | 0 (1) | 0.00 (0.002) |  |
| Female-biased only in InR^DN^ comparison | 1,650 (3,587) | 19.63 (9.09) | 537 (1,282) | 6.39 (3.25) |  |
| Male-biased | 87 (105) | 1.04 (0.27) | 10 (17) | 0.12 (0.04) |  |
| Male-biased only in control comparison | 111 (144) | 1.32 (0.36) | 21 (39) | 0.25 (0.10) |  |
| Male-biased only in InR^DN^ comparison | 1,009 (1,642) | 12.00 (4.16) | 206 (331) | 2.45 (0.84) |  |
| Male-biased in control comparison,  Female-biased in InR^DN^ comparison | 16 (22) | 0.19 (0.06) | 2 (2) | 0.02 (0.01) |  |
|  |  |  |  |  |  |

Note, in some cases the different exons of a gene corresponded to different categories. In these cases genes were flagged as belonging to multiple categories. For the between sex comparisons n = 2,748 genes were included in only one category at FDR < 0.01 and n = 232 genes were placed in more than one category at FDR < 0.01. For ln-fold change > 0.69 or < -0.69 and FDR < 0.01, n = 790 genes were included in only one category at FDR < 0.01 and n = 22 genes were placed in more than one category at FDR < 0.01.
