# Supplementary material for: Perturbation of IIS/TOR signaling alters the landscape of sex-differential gene expression in Drosophila
Source: BMC Genomics. 2018 Dec 10;19:893. doi: 10.1186/s12864-018-5308-3 (PMC6288939; doi:10.1186/s12864-018-5308-3)

Figure S3: Distribution of exon-level estimates of expression differences within males and females. For each category in Figure 2 (see legend) the distribution of the estimate of the expression difference (as the difference in ln-RPKM) is shown as a vertical histogram. Positive values result from lower expression with reduced insulin signaling, relative to control (downregulation in InR^{DN}) and negative values result from increased expression with reduced insulin signaling, relative to control (upregulation in InR^{DN}). Outlier box plots show the median (vertical line), interquartile range (within the box), and the mean and upper and lower 95% confidence intervals (diamond). The densest region of the data is indicated by the bracket on the left side of the box. **A.** Expression differences within females and **B.** Expression differences within males.

A. Within Females

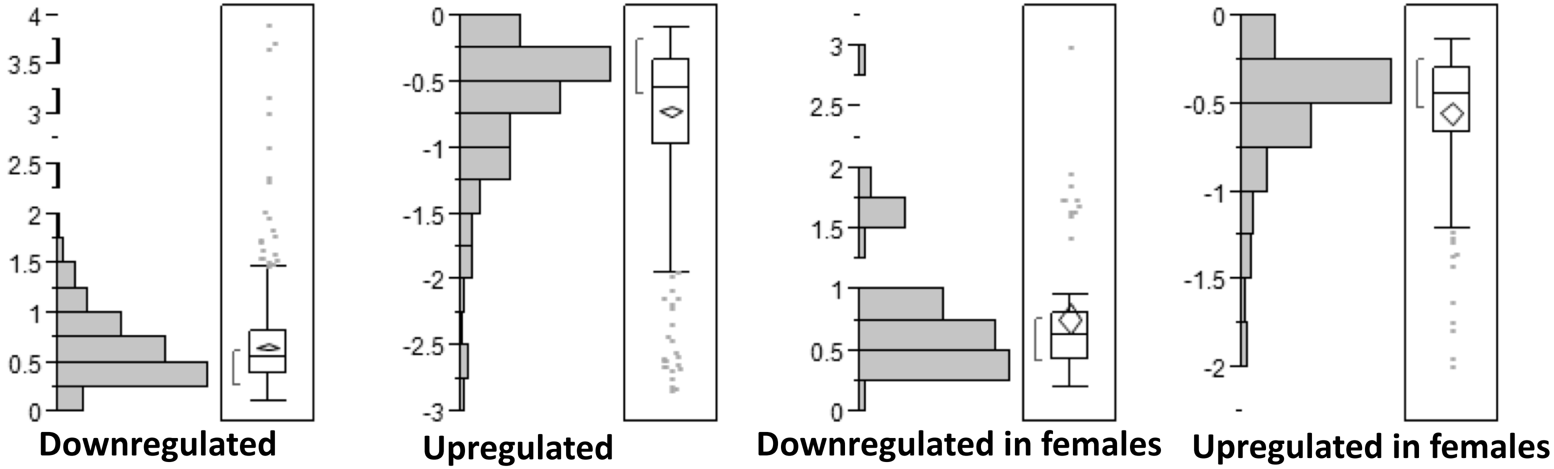

B. Within Males

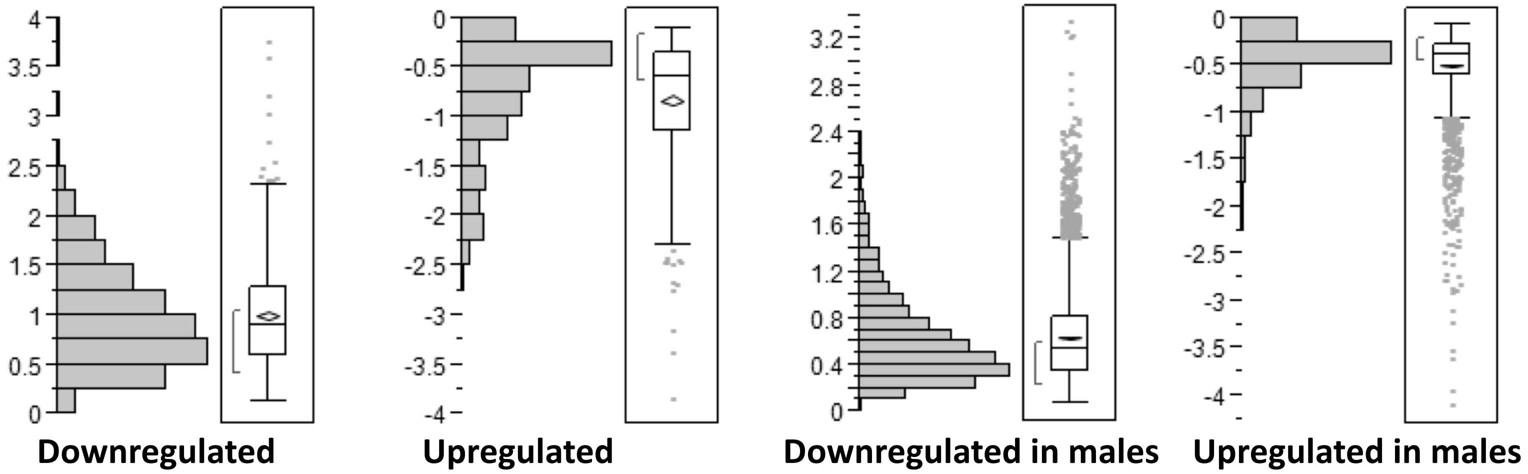

Supplement: Supplementary file 6 — Figure S3: Within sex comparison: Histograms. (PDF 618 kb) [file 12864_2018_5308_MOESM6_ESM.pdf]
