# Supplementary material for: Perturbation of IIS/TOR signaling alters the landscape of sex-differential gene expression in Drosophila
Source: BMC Genomics. 2018 Dec 10;19:893. doi: 10.1186/s12864-018-5308-3 (PMC6288939; doi:10.1186/s12864-018-5308-3)

Figure S4: Distribution of exon-level estimates of expression differences between males and females. For each category in Figure 5 (see legend) the distribution of the estimate of the expression difference (as the difference in ln-RPKM) is shown as a vertical histogram. Positive values result from lower expression in males, relative to females (female-biased) and negative values result from increased expression in males, relative to females (male-biased). Outlier box plots show the median (vertical line), interquartile range (within the box), and the mean and upper and lower 95% confidence intervals (diamond). The densest region of the data is indicated by the bracket on the left side of the box. **A.** Expression differences between males and females in control conditions and **B.** Expression differences between males and females with exposure to the drug resulting in expression of InR^{DN} and reduced insulin signaling.

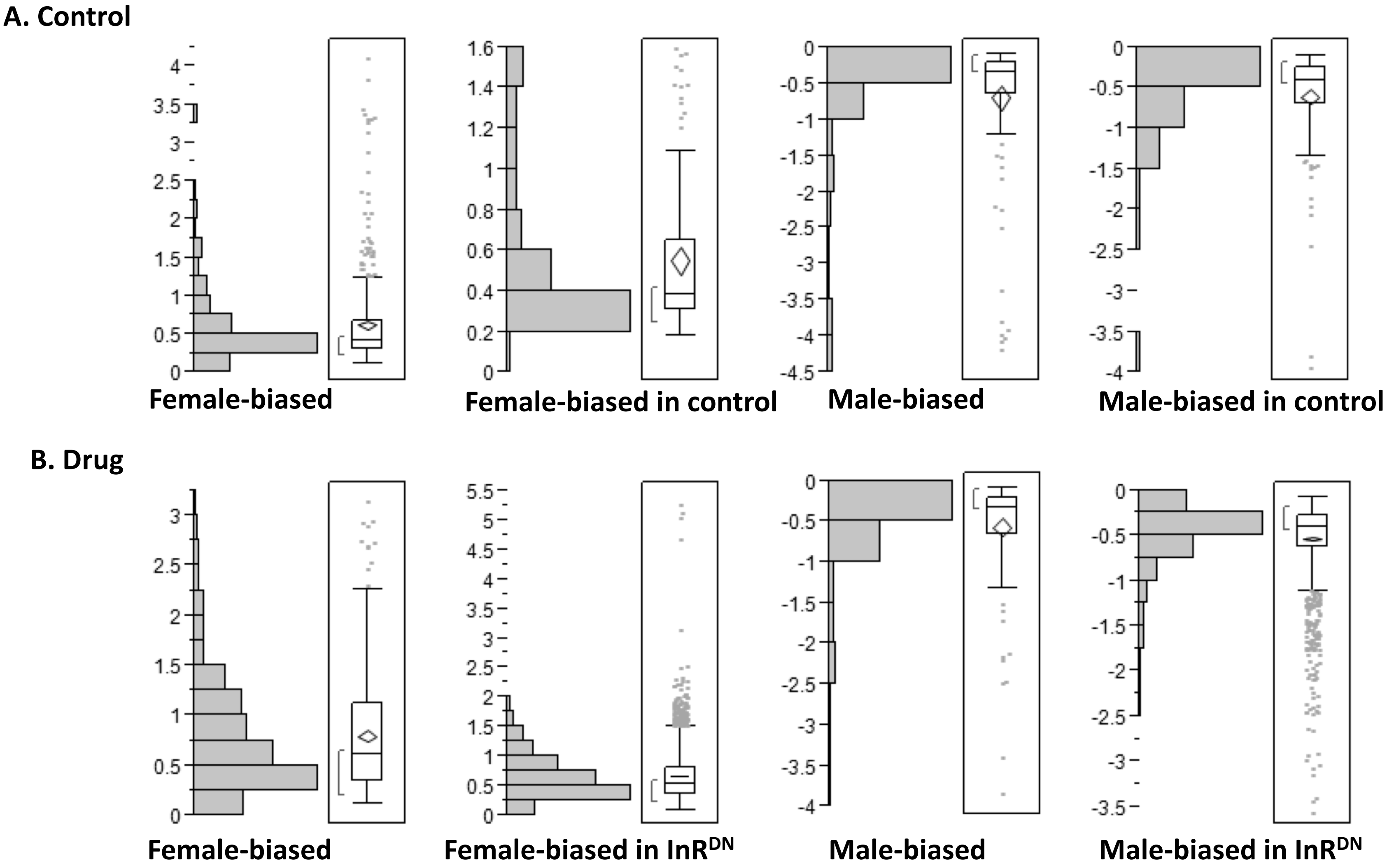

Supplement: Supplementary file 9 — Figure S4: Between sex comparison: Histograms. (PDF 404 kb) [file 12864_2018_5308_MOESM9_ESM.pdf]
