# Supplementary material for: Perturbation of IIS/TOR signaling alters the landscape of sex-differential gene expression in Drosophila
Source: BMC Genomics. 2018 Dec 10;19:893. doi: 10.1186/s12864-018-5308-3 (PMC6288939; doi:10.1186/s12864-018-5308-3)

Figure S5: Reduced InR signaling impacts sex-differential expression.
The GO Biological Process category (top) and KEGG pathway (bottom) with the most significant sex changes are shown for control (left) and InR^{DN}-expressing (right). The p-value is indicated by the red-purple scale, with red being most significant. The number of genes that were considered in the analyses is plotted on the X-axis. Pathways and groups that are uniquely significant in one comparison are indicated by green font.

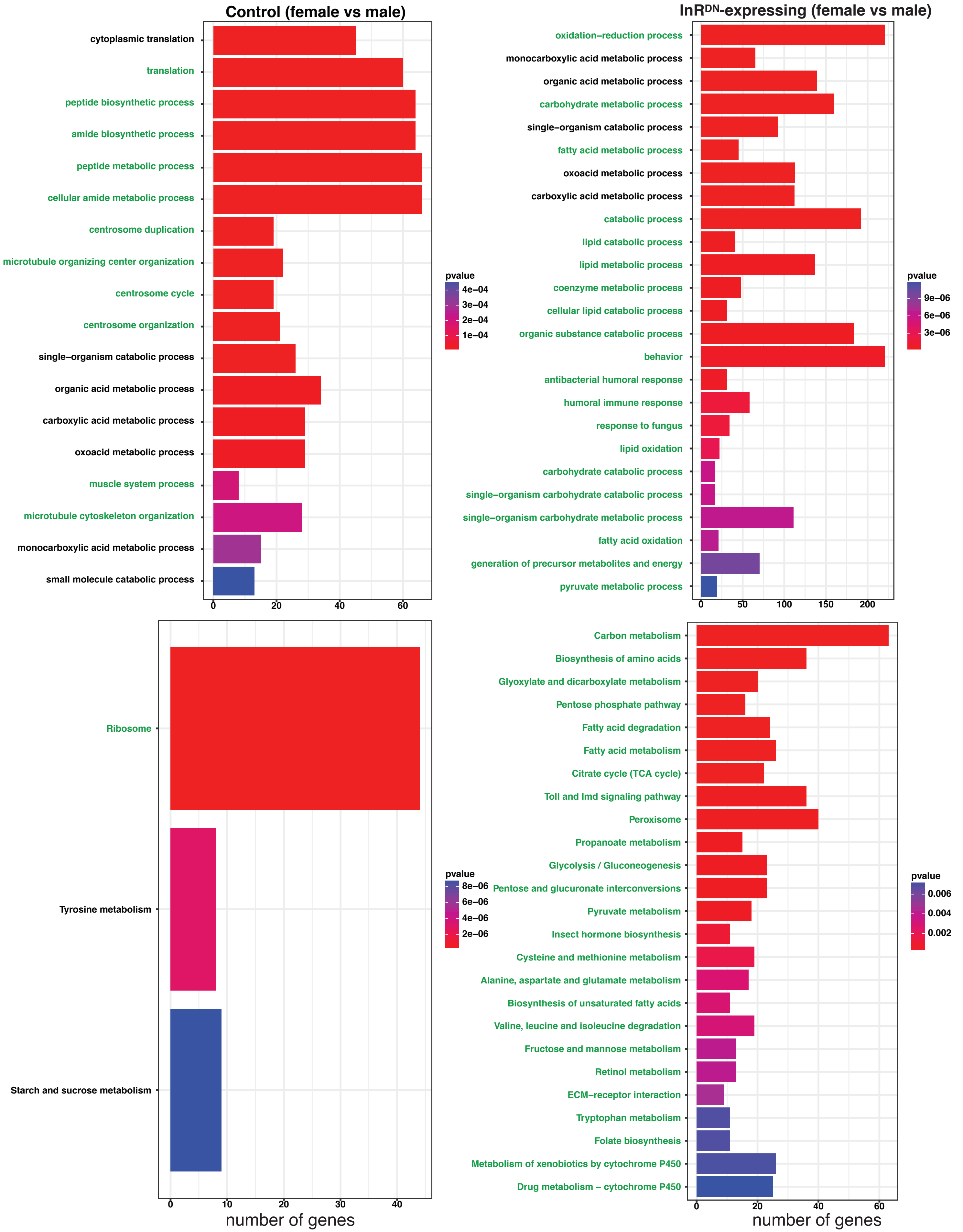

Supplement: Supplementary file 10 — Figure S5: GO and KEGG enrichments. (PDF 1064 kb) [file 12864_2018_5308_MOESM10_ESM.pdf]
