# Supplementary material for: Perturbation of IIS/TOR signaling alters the landscape of sex-differential gene expression in Drosophila
Source: BMC Genomics. 2018 Dec 10;19:893. doi: 10.1186/s12864-018-5308-3 (PMC6288939; doi:10.1186/s12864-018-5308-3)

Figure S6a: Expression differences for selected genes. Bar charts show mean estimated expression (RPKM) for all detected exons of selected example genes (*doublesex*, *fruitless*, *Juvenile hormone epoxide hydrolase 1*, and *spinster*). Error bars represent one standard deviation. X-axis labels are FlyBase exon names (FB5.51 annotation). The expression in females under control conditions is shown in blue and with InR^{DN} expression in orange. The expression in males under control conditions is shown in grey and with InR^{DN} expression in yellow.

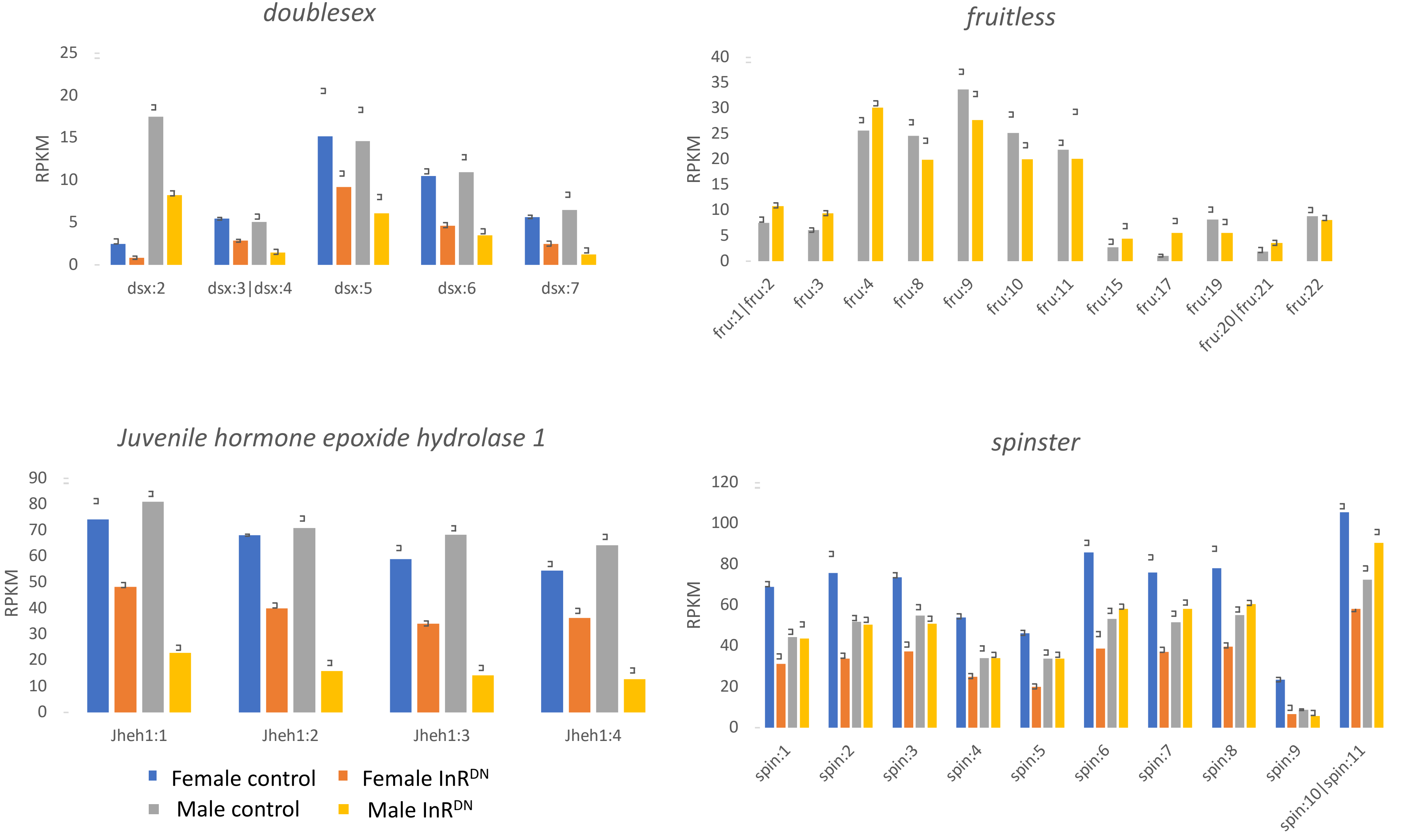

Supplemental Figure 6b: Expression differences for selected genes. Bar charts show mean estimated expression (RPKM) for all detected exons of selected example genes (*ilp2, ilp3, ilp5 and ilp6 genes*). Error bars represent one standard deviation. X-axis labels are FlyBase exon names (FB5.51 annotation). The expression in females under control conditions is shown in blue and with InR^{DN} expression in orange. The expression in males under control conditions is shown in grey and with InR^{DN} expression in yellow.

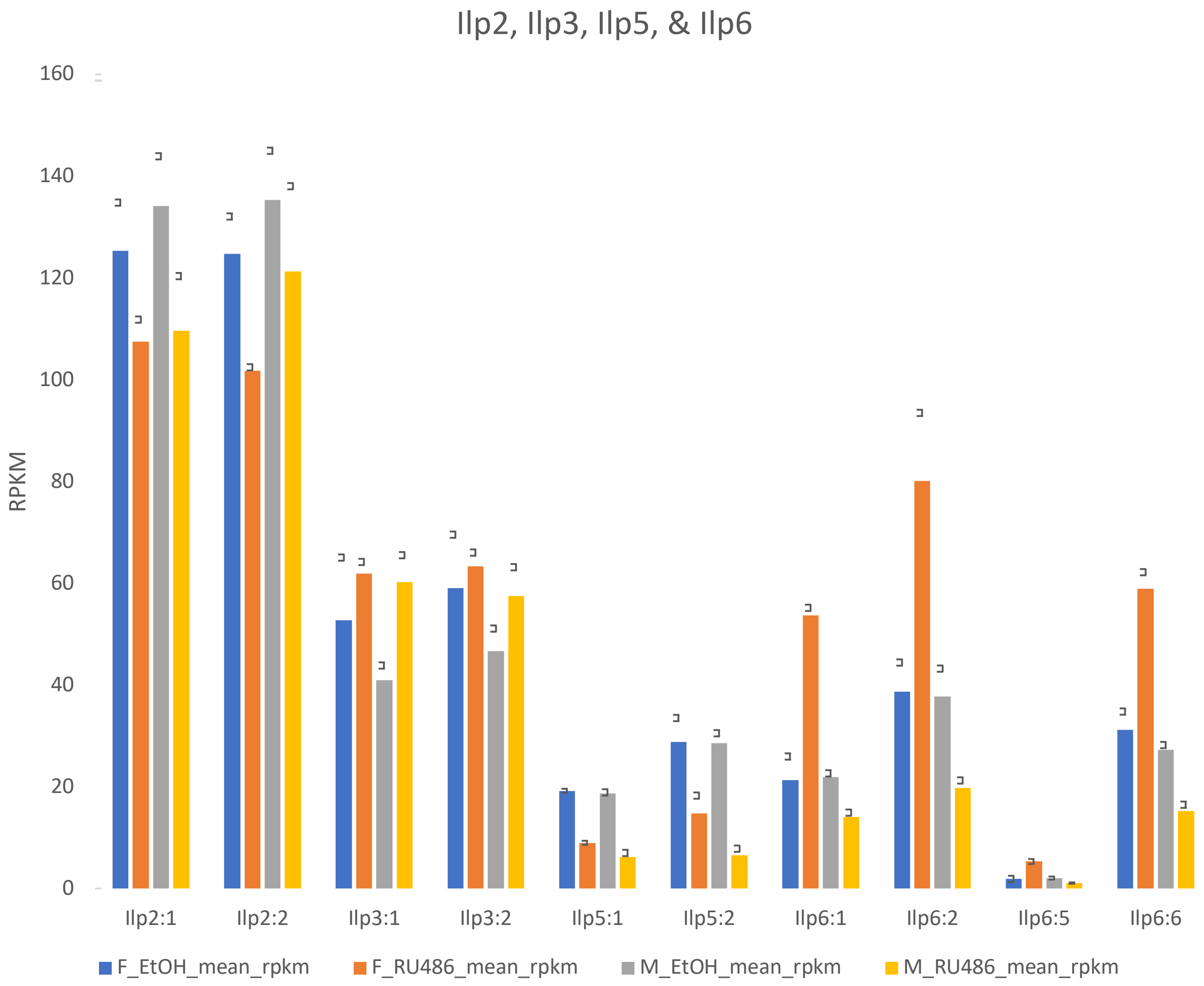

Supplement: Supplementary file 11 — Figure S6: Bar charts showing expression for individual exons for genes. (PDF 547 kb) [file 12864_2018_5308_MOESM11_ESM.pdf]
